# Acceptance of Anti-Retroviral Therapy among Patients Infected with HIV and Tuberculosis in Rural Malawi Is Low and Associated with Cost of Transport

**DOI:** 10.1371/journal.pone.0000121

**Published:** 2006-12-27

**Authors:** Rony Zachariah, Anthony David Harries, Marcel Manzi, Patrick Gomani, Roger Teck, Mit Phillips, Peter Firmenich

**Affiliations:** 1 Medecins sans Frontieres, Medical Department (Operational Research), Brussels Operational Center, Brussels, Belgium; 2 Ministry of Health, HIV Care and Support, Lilongwe, Malawi; 3 Ministry of Health and Population, Thyolo District Health Services, Thyolo, Malawi; 4 Medecins sans Frontieres, Thyolo, Malawi; 5 Medecins sans Frontieres, Access to Health, Brussels, Belgium; 6 Medecins sans Frontieres, Operations Department, Brussels Operational Center, Luxembourg; University of Sydney, Australia

## Abstract

**Background:**

A study was conducted among newly registered HIV-positive tuberculosis (TB) patients systematically offered anti-retroviral treatment (ART) in a district hospital in rural Malawi in order to a) determine the acceptance of ART b) conduct a geographic mapping of those placed on ART and c) examine the association between “cost of transport” and ART acceptance.

**Methodology/Principal Findings:**

A retrospective cross-sectional analysis was performed on routine program data for the period of February 2003 to July 2004. Standardized registers and patient cards were used to gather data. The place of residence was used to determine road distances to the Thyolo district hospital. Cost of transport from different parts of the district was based on the known cost for public transport to the road-stop closest to the patient's residence. Of 1,290 newly registered TB patients, 1,003(78%) underwent HIV-testing of whom 770 (77%) were HIV-positive. 742 of these individuals (pulmonary TB = 607; extra-pulmonary TB = 135) were considered eligible for ART of whom only 101(13.6%) accepted ART. Cost of transport to the hospital ART site was significantly associated with ART acceptance and there was a linear trend in association between cost and ART acceptance (X^2^ for trend = 25.4, P<0.001). Individuals who had to pay 50 Malawi Kwacha (1 United States Dollar = 100 Malawi Kwacha, MW) or less for a one-way trip to the Thyolo hospital were four times more likely to accept ART than those who had to pay over 100 MW (Adjusted Odds ratio = 4.0, 95% confidence interval: 2.0–8.1, P<0.001).

**Conclusions/Significance:**

ART acceptance among TB patients in a rural district in Malawi is low and associated with cost of transport to the centralized hospital based ART site. Decentralizing the ART offer from the hospital to health centers that are closer to home communities would be an essential step towards reducing the overall cost and burden of travel.

## Introduction

Tuberculosis (TB) is one of the most common causes of morbidity and mortality in HIV positive populations [1], [2] and TB often brings the HIV-positive individual to medical attention. Since TB patients constitute a readily identifiable group in the health system for HIV-testing, they thus offer an opportunity for introducing HIV-related interventions including antiretroviral treatment (ART) [3].

Malawi, a small resource-poor country in southern Africa, has an estimated national HIV prevalence rate of 9%,[4] and 77% of new patients registered with TB are co-infected with HIV [5]. The Malawi ART guidelines [6] recommend that HIV-positive individuals assessed to be in World Health Organization (WHO) clinical stages III and IV are eligible for ART. As HIV-positive individuals with pulmonary TB (PTB) are classified as WHO stage III and those with extrapulmonary TB (EPTB) as WHO stage IV, all such TB patients are considered eligible for ART in Malawi.

In Thyolo district, rural southern Malawi, ART was initiated in the main public hospital of the district in early 2003. However, despite a systematic offer of ART to all HIV-positive TB patients, acceptance of ART in this group was found to be at a low 13% [7].

It is likely that multiple factors might be influencing ART acceptance in HIV-positive TB patients in this setting, but these are at present not well characterized. We hypothesized that the “cost of transport” to a single, centralized ART delivery site in a poor rural setting where public transport networks are poorly developed is likely to be one of such factors, and a possible barrier for ART acceptance.

We conducted a study in Thyolo in order to a) determine the acceptance of ART among newly registered HIV-positive TB patients; b) conduct a geographic mapping of those placed on ART; and c) examine the association between “cost of transport” and ART acceptance.

## Methods

### Study setting and management of TB

This study was conducted between February 2003 and July 2004 in Thyolo district, a rural region in Southern Malawi with approximately 500 000 inhabitants. All newly registered HIV-positive TB patients presenting to the main public hospital in the district (Thyolo hospital) were involved in the study. TB patients are systematically offered counseling and HIV testing, using rapid whole blood testing kits, according to the WHO strategy II for HIV antibody testing [8]. Cotrimoxazole prophylaxis is offered to all HIV-positive TB patients at a dose of 960 mg daily provided there are no contraindications.

Anti-TB treatment is based on national guidelines and has been described before [6]. In the initial phase of treatment, patients are admitted to the hospital TB wards for 2 weeks during which they receive directly observed anti-TB treatment. Patients are then allowed to go home, and receive their remaining 6 weeks of initial phase anti-TB treatment under supervision at a health center, or from a guardian. A guardian in this context refers to a family member or someone in the neighbourhood who will ensure that the patient does take the prescribed treatment. They are then placed on the six-month continuation phase of treatment where they receive isoniazid and ethambutol on a monthly basis from health centers (decentralized sites). Cotrimoxazole is also provided and made available at all health centers for continued prophylaxis.

### ART for HIV-positive TB patients

The first line ART regimen in Malawi is a fixed dose combination of Stavudine (d4T), Lamivudine (3TC) and Nevirapine (NVP) [6]. Because of concern about drug interactions between rifampicin and nevirapine, ART is offered to all HIV-positive TB patients only after completing the initial two months of anti-TB treatment.

HIV-positive TB patients are systematically informed of the offer of ART and requested to return to the hospital HIV-ART clinic eight weeks after starting anti-TB treatment in conjunction with a patient guardian to prepare for ART initiation. Once started on ART, patients are reviewed back at the HIV-ART clinic after two weeks. From then on, provided there are no side effects, patients are seen monthly at the ART clinic and given drugs every 28 days indefinitely. ART and anti-TB treatment are offered free of charge in Thyolo and at the time of the study the Thyolo hospital was the only ART delivery site in the district.

### Data collection and statistical analysis

The counseling, TB and ART registers [9] as well as patient cards were used to gather basic socio-demographic data, as well as information on HIV testing, type of TB and ART uptake.

The place of residence as indicated on the TB patient card and register was used to determine road distances to the Thyolo district hospital. Cost of transport from different parts of the district were based on the known cost for public transport to the road-stop closest to the patients residence. Two traditional authorities namely Khwethmule and Thukuta traditional did not have TB treatment units. Data on ART acceptance for these two areas was thus included with that of Nsabwe, Changata, and Mpuka depending on where the patient was being followed for TB treatment.

Newly registered HIV-positive TB patients placed on ART were designated as the dependent variable for identifying potential associations. The measures of risk were determined by crude odds ratios (OR) and adjusted odds ratios (adjusted OR). Odds ratios were adjusted using multi-variate logistic regression, and all related *P*-values are based on the wald test. The χ^2^ test for trend was used to test for linear trends. The level of significance was set at *P* = 0.05 or less and 95% confidence intervals were used throughout.

## Results

### Characteristics of the study population

**Figure 1:**
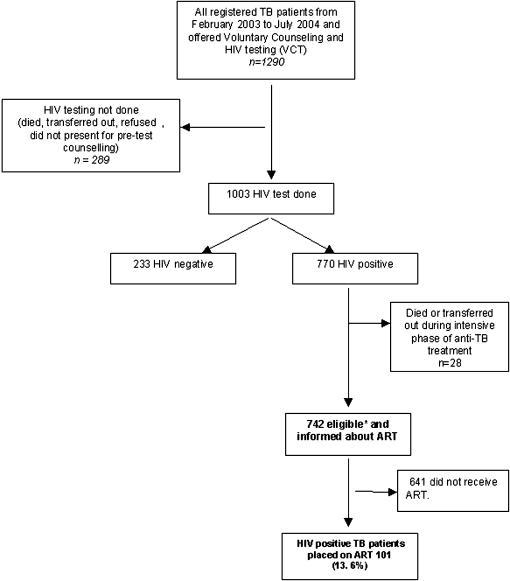
Uptake of antiretroviral treatment among tuberculosis patients. *Individuals who had completed their initial phase (2 months) of anti-TB treatment and were supposed to start ART during the period April 2003 to September 2004.

There were 1290 patients registered for TB during the study period **(** **FIGURE 1** **)**. HIV testing was done in 1003 (78%) of whom 770(77%) were found to be HIV positive. During the intensive phase of anti-TB treatment, 19 HIV positive TB patients died and 9 were transferred out of the district; these individuals are excluded from the study. Of the 742 HIV-positive TB patients who completed the intensive phase of anti-TB treatment and were considered eligible for ART, there were 420 women and 322 men (median age: 32 years, range 2-70 years). 376 (51%) patients were married while 366 were single (unmarried, divorced or widowed ). The great majority (76%) of TB patients were subsistence farmers while tea and coffee estate employees, petty traders and students comprised the rest. There were 607 (82%) individuals with pulmonary TB (smear-positive or negative) and 135 individuals with EPTB. 716 (93%) of the 770 HIV-positive TB patients were placed on cotrimoxazole prophylaxis.

### Uptake of ART among HIV-positive TB patients

Of 742 HIV-positive TB patients who completed the intensive phase of anti-TB treatment and were offered ART, only 101(13.6%) were started on ART **(** **FIGURE 1** **)**.

### Geographic mapping of HIV positive TB patients placed on ART in Thyolo district

**Figure 2:**
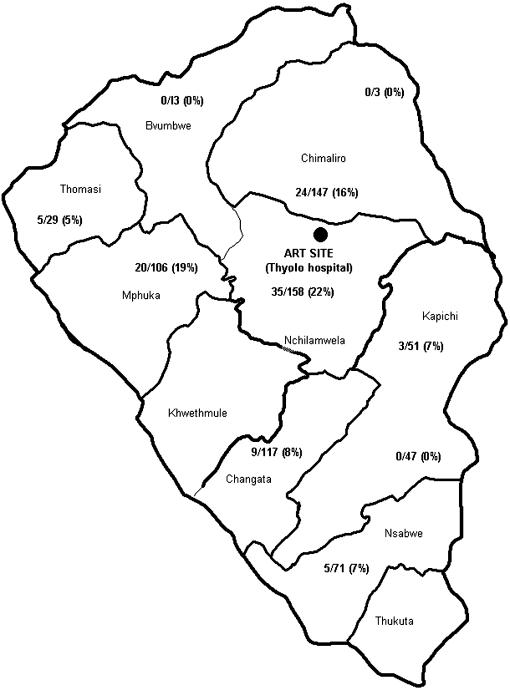
Geographic distribution of tuberculosis cases placed on ART. •ART delivery site, Thyolo hospital. Numbers and percentages (%) represent the proportion of HIV-positive TB patients placed on ART.

**FIGURE 2** shows the single ART delivery site (Thyolo hospital), the geographic distribution of HIV-positive TB patients within traditional authorities (or from borderline areas), and the respective proportions who were placed on ART. The median distance that HIV-positive TB patients had to travel to get to the Thyolo hospital was 22 km (range 5 to 65 km) and the median cost for transport was 70 Malawi kwacha (range 30 to 270 kwacha, 1 United States Dollar, USD = 100 Malawi Kwacha). 79 (78%) of 101 TB patients placed on ART came from three traditional authorities that lie within the proximity of the ART delivery site and where cost of transport was less than 70 Malawi kwacha.

### Factors associated with ART uptake

**TABLE 1** shows the association between various factors and ART acceptance among HIV-positive TB patients. Cost of transport to the hospital ART site was significantly associated with ART acceptance and there was a linear trend in association between cost and ART acceptance (X^2^ for trend = 25.4 *P* = <0.001). Individuals who had to pay 50 Malawi Kwacha (half a United States dollar) or less for a one way trip to the Thyolo hospital were four times more likely to take up the offer of ART than those who had to pay 100 (or more) Malawi Kwacha (≥1 USD).

**Table 1:**
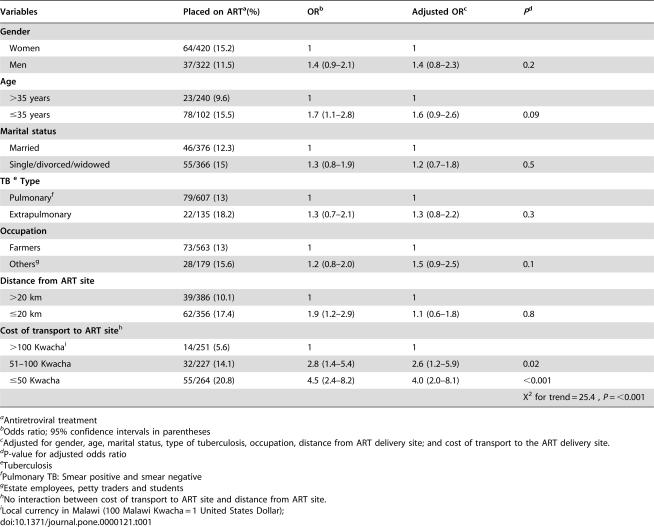
Factors associated with uptake of anti-retroviral treatment among tuberculosis (TB) patients.

Variables	Placed on ART^a^(%)	OR^b^	Adjusted OR^c^	*P* d
**Gender**				
Women	64/420 (15.2)	1	1	
Men	37/322 (11.5)	1.4 (0.9–2.1)	1.4 (0.8–2.3)	0.2
**Age**				
>35 years	23/240 (9.6)	1	1	
≤35 years	78/102 (15.5)	1.7 (1.1–2.8)	1.6 (0.9–2.6)	0.09
**Marital status**				
Married	46/376 (12.3)	1	1	
Single/divorced/widowed	55/366 (15)	1.3 (0.8–1.9)	1.2 (0.7–1.8)	0.5
**TB e Type**				
Pulmonary^f^	79/607 (13)	1	1	
Extrapulmonary	22/135 (18.2)	1.3 (0.7–2.1)	1.3 (0.8–2.2)	0.3
**Occupation**				
Farmers	73/563 (13)	1	1	
Others^g^	28/179 (15.6)	1.2 (0.8–2.0)	1.5 (0.9–2.5)	0.1
**Distance from ART site**				
>20 km	39/386 (10.1)	1	1	
≤20 km	62/356 (17.4)	1.9 (1.2–2.9)	1.1 (0.6–1.8)	0.8
**Cost of transport to ART site** h				
>100 Kwacha^i^	14/251 (5.6)	1	1	
51–100 Kwacha	32/227 (14.1)	2.8 (1.4–5.4)	2.6 (1.2–5.9)	0.02
≤50 Kwacha	55/264 (20.8)	4.5 (2.4–8.2)	4.0 (2.0–8.1)	<0.001
				X^2^ for trend = 25.4 , *P* = <0.001

a Antiretroviral treatment

b Odds ratio; 95% confidence intervals in parentheses

c Adjusted for gender, age, marital status, type of tuberculosis, occupation, distance from ART delivery site; and cost of transport to the ART delivery site.

d P-value for adjusted odds ratio

e Tuberculosis

f Pulmonary TB: Smear positive and smear negative

g Estate employees, petty traders and students

h No interaction between cost of transport to ART site and distance from ART site.

i Local currency in Malawi (100 Malawi Kwacha = 1 United States Dollar);

## Discussion

This study shows that ART acceptance among TB patients in a rural district in Malawi is low and associated with cost of transport to the centralized hospital based ART site. The higher the cost of transport to the hospital based ART delivery site, the less probable it is that a TB patient accepts ART.

Distance to the hospital facility per-se was not significantly associated with ART uptake and this is likely linked to the fact that cost of transport is much more related to the presence (or not) of public transport networks. The relationship between distance and cost of transport is not proportional, because where public transport is well developed, the cost for a given distance would be relatively less and vise-versa.

The great majority of TB patients in our setting are poor subsistence farmers earning less than 4 USD per week [10]. Evidence from other similar settings in Malawi has revealed that access costs linked to establishing a TB diagnosis and starting treatment alone amounts to seven months or more of annual income for such individuals [11]. Thus, the payment of even just one 1 USD for a return trip to an ART site will account for at least 25% of weekly net-revenue, and the financial predicament of TB patients at the time of starting ART might mean that they are simply too impoverished to afford such a sum linked to accessing ART. There is also recent evidence from the ART-Link collaboration that shows that mortality of HIV-1 infected patients in the first year of antiretroviral therapy was increased in ART-programs that charged user fees [12].

In contrast to the current centralized offer of ART, cotrimoxazole prophylaxis has been decentralized along with anti-TB treatment and the drug is made available in all health centers. Patients thus receive their monthly supply of cotrimoxazole along with anti-TB drugs at sites close to their home communities. The overall burden and particularly cost of transport is thus minimized. In contrast to ART, the uptake of cotrimoxazole in this cohort of patients is 93%, and other studies in the same setting have demonstrated that adherence to cotrimoxazole both during and after anti-TB treatment is close to 94% [10], [13].

One of the logical ways forward in trying to minimize the out of pocket transport expenses associated with accessing ART from a centralized site would be to decentralize ART to health centers in a similar manner as has been done for anti-TB treatment and cotrimoxazole. This would have a number of important advantages:

First, from the patient perspective, the overall travel related burden would be reduced. This would be of particular relevance to ill TB patients who live in relatively remote areas and are thus unable to make multiple journeys either due to cost or other burdens of travel [l0,14].

Second, decentralization of ART-initiation to health centers would mean integration of both TB and HIV/AIDS services within one health facility and involvement of the same team with patient care. Such an approach would be more holistic and may influence the patient's perception of the link between the two diseases, which in turn may positively influence ART-seeking behavior among TB patients.

Third, the great majority of TB patients tend to feel better after completing the two first months of the intensive phase of anti-TB treatment. These patients would probably prefer to continue treatment in health centers close to their communities [15], [16]. This patient friendly approach based around health centers, if coupled with supportive community involvement, is also likely to be more cost-effective in the long run [17], [18].

Finally, from a district hospital perspective, the workload on a centralized hospital-based ART clinic will be progressively reduced as ART initiation becomes progressively decentralized to health centres. Despite these obvious advantages, decentralizing the initiation of ART to health centers raises a number of operational implications that will need to be addressed. First, selected health centers would need to have appropriate infrastructure to be able to run a number of key activities: counseling, clinical management of opportunistic infections, support and education of patients for ART, registration and initiation of ARV therapy and follow-up of patients. Second, there has to be adequate human resources to perform these additional tasks. Currently only 50% of available posts in the Ministry of Health in Malawi are filled [17] and it is estimated that the minimum additional staff requirement to initiate first-line ART would include a full-time clinical officer (or nurse), a counselor and a clerk [6]. Health centers might need time to build up these additional human resources. An interim solution , might be to introduce a mobile district based “ART initiation team”. Third, health staff will need to be trained and certified as being competent to manage ART. Finally, registration and monitoring tools will also have to be placed at health centers and a mechanism put in place to ensure regular ART drug supply, drug security and supervision.

The strengths of this study are that an impressive proportion of TB patients underwent HIV-testing and were included in the analysis, and as the data comes from a routine TB program setting the results reflect the reality on the ground. However, this study was only designed to examine the association between cost of transport and ART acceptance and there are a number if other relevant questions that cannot be answered here and which merit further specific research: a) is the rather long (8 week) waiting time between the initiation of anti-TB treatment and ART influencing ART acceptance ? b) are TB patients who feel better with anti-TB treatment and cotrimoxazole not appreciating the extra need for ART? c) are patients in the district hospital not being educated enough about HIV/AIDS to realize the importance of ART? and d) are there other unrecognized barriers to ART uptake ?

Whatever might be the eventual answers to these and other questions, in the meantime, decentralizing the ART offer from the hospital to health centers that are closer to home communities would be an essential step towards reducing the overall cost and burden of travel. Despite the operational challenges, and hard work ahead, this would seem a worthwhile goal to strive for!
